# Supplementary material for: Transcatheter aortic valve implantation versus conservative management for severe aortic stenosis in real clinical practice
Source: PLoS One. 2019 Sep 26;14(9):e0222979. doi: 10.1371/journal.pone.0222979 (PMC6762145; doi:10.1371/journal.pone.0222979)
Supplement: S1 Text — (DOCX) [file pone.0222979.s001.docx]

**S1 Text. Study Organization**

**Study Organization of CURRENT AS registry**

**Clinical Event Committee:**

Hirotoshi Watanabe, Kenji Nakatsuma, Tomoki Sasa

**Statistical Analysis:**

Takeshi Morimoto

**Principal Investigators:**

Takeshi Kimura, Ryuzo Sakata

**Study Organization of K-TAVI registry**

**Principal Investigators:**

Takeshi Kimura
