# Supplementary material for: Transcatheter aortic valve implantation versus conservative management for severe aortic stenosis in real clinical practice
Source: PLoS One. 2019 Sep 26;14(9):e0222979. doi: 10.1371/journal.pone.0222979 (PMC6762145; doi:10.1371/journal.pone.0222979)
Supplement: S2 Text — (DOCX) [file pone.0222979.s002.docx]

**S2 Text. List of participating centers and investigators.**

**List of participating centers and investigators for the CURRENT AS registry**

**Cardiology**

**Kyoto University Graduate School of Medicine:** Takeshi Kimura, Tomohiko Taniguchi,

Hiroki Shiomi, Naritatsu Saito, Masao Imai, Junichi Tazaki, Toshiaki Toyota,

Hirooki Higami, Tetsuma Kawaji

**Kokura Memorial Hospital:** Kenji Ando, Shinichi Shirai, Kengo Kourai, Takeshi Arita, Shiro Miura

**Shimada Municipal Hospital:** Takeshi Aoyama, Norio Kanamori

**Shizuoka City Shizuoka Hospital:** Tomoya Onodera, Koichiro Murata

**Kobe City Medical Center General Hospital:** Yutaka Furukawa, Takeshi Kitai

**Kurashiki Central Hospital:** Kazushige Kadota, Yuichi Kawase, Keiichiro Iwasaki, Hiroshi Miyawaki, Ayumi Misao, Akimune Kuwayama, Masanobu Ohya, Takenobu Shimada, Hidewo Amano

**Tenri Hospital:** Yoshihisa Nakagawa, Chisato Izumi, Makoto Miyake, Masashi Amano, Yusuke Takahashi, Yusuke Yoshikawa, Shunsuke Nishimura, Maiko Kuroda

**Nara Hospital, Kinki University Faculty of Medicine:** Manabu Shirotani, Hirokazu Mitsuoka

**Mitsubishi Kyoto Hospital:** Shinji Miki, Tetsu Mizoguchi, Masashi Kato, Takafumi Yokomatsu, Akihiro Kushiyama, Hidenori Yaku, Toshimitsu Watanabe

**Kinki University Hospital:** Shunichi Miyazaki, Yutaka Hirano

**Kishiwada City Hospital:** Mitsuo Matsuda, Shintaro Matsuda, Sachiko Sugioka

**Osaka Red Cross Hospital:** Tsukasa Inada, Kazuya Nagao, Naoki Takahashi, Kohei Fukuchi

**Koto Memorial Hospital:** Tomoyuki Murakami, Hiroshi Mabuchi, Teruki Takeda, Tomoko Sakaguchi, Keiko Maeda, Masayuki Yamaji, Motoyoshi Maenaka, Yutaka Tadano

**Shizuoka General Hospital:** Hiroki Sakamoto, Yasuyo Takeuchi, Makoto Motooka

**Nishikobe Medical Center:** Hiroshi Eizawa, Keiichiro Yamane, Mitsunori Kawato, Minako Kinoshita, Kenji Aida

**Japanese Red Cross Wakayama Medical Center:** Takashi Tamura, Mamoru Toyofuku, Kousuke Takahashi, Euihong Ko

**National Hospital Organization Kyoto Medical Center:** Masaharu Akao, Mitsuru Ishii, Nobutoyo Masunaga, Hisashi Ogawa, Moritake Iguchi, Takashi Unoki, Kensuke Takabayashi, Yasuhiro Hamatani, Yugo Yamashita

**The Tazuke Kofukai Medical Research Institute, Kitano Hospital:** Moriaki Inoko, Eri Minamino-Muta, Takao Kato

**Hikone Municipal Hospital:** Yoshihiro Himura, Tomoyuki Ikeda

**Kansai Electric Power Hospital:** Katsuhisa Ishii, Akihiro Komasa

**Hyogo Prefectural Amagasaki General Medical Center:** Yukihito Sato, Kozo Hotta, Shuhei Tsuji

**Rakuwakai Otowa Hospital:** Yuji Hiraoka, Nobuya Higashitani

**Saiseikai Noe Hospital:** Ichiro Kouchi, Yoshihiro Kato

**Shiga Medical Center for Adults:** Shigeru Ikeguchi, Yasutaka Inuzuka, Soji Nishio, Jyunya Seki

**Hamamatsu Rosai Hospital:** Eiji Shinoda, Miho Yamada, Akira Kawamoto, Chiyo Maeda

**Japanese Red Cross Otsu Hospital:** Takashi Konishi, Toshikazu Jinnai, Kouji Sogabe, Michiya Tachiiri, Yukiko Matsumura, Chihiro Ota

**Hirakata Kohsai Hospital:** Shoji Kitaguchi, Yuko Morikami

**Cardiovascular Surgery**

**Kyoto University Graduate School of Medicine:** Ryuzo Sakata, Kenji Minakata, Kenji Minatoya

**Kokura Memorial Hospital:** Michiya Hanyu

**Shizuoka City Shizuoka Hospital:** Fumio Yamazaki

**Kobe City Medical Center General Hospital:** Tadaaki Koyama

**Kurashiki Central Hospital:** Tatsuhiko Komiya

**Tenri Hospital:** Kazuo Yamanaka

**Nara Hospital, Kinki University Faculty of Medicine:** Noboru Nishiwaki

**Mitsubishi Kyoto Hospital:** Hiroyuki Nakajima, Motoaki Ohnaka, Hiroaki Osada, Katsuaki Meshii

**Kinki University Hospital:** Toshihiko Saga

**Kishiwada City Hospital:** Masahiko Onoe

**Osaka Red Cross Hospital:** Shogo Nakayama

**Shizuoka General Hospital:** Genichi Sakaguchi

**Japanese Red Cross Wakayama Medical Center:** Atsushi Iwakura

**National Hospital Organization Kyoto Medical Center:** Kotaro Shiraga

**The Tazuke Kofukai Medical Research Institute, Kitano Hospital:** Koji Ueyama

**Hyogo Prefectural Amagasaki General Medical Center:** Keiichi Fujiwara

**Rakuwakai Otowa Hospital:** Atsushi Fukumoto

**Shiga Medical Center for Adults:** Masaki Park

**Hamamatsu Rosai Hospital:** Junichiro Nishizawa

**Japanese Red Cross Otsu Hospital:** Mitsuru Kitano

**List of participating centers and investigators for K-TAVI registry**

**Cardiology**

**Kyoto University Graduate School of Medicine:** Takeshi Kimura, Naritatsu Saito,

Tomohiko Taniguchi

**Kokura Memorial Hospital:** Shinichi Shirai

**Shizuoka City Shizuoka Hospital:** Koichiro Murata

**Kobe City Medical Center General Hospital:** Natsuhiko Ehara

**Kurashiki Central Hospital:** Tsuyoshi Goto, Yasushi Fuku

**Tenri Hospital:** Yoshihisa Nakagawa, Toshihiro Tamura

**Cardiovascular Surgery**

**Kyoto University Graduate School of Medicine:** Kenji Minakata, Shinya Takimoto

**Kokura Memorial Hospital:** Michiya Hanyu

**Shizuoka City Shizuoka Hospital:** Fumio Yamazaki

**Kobe City Medical Center General Hospital:** Tadaaki Koyama

**Kurashiki Central Hospital:** Tatsuhiko Komiya

**Tenri Hospital:** Kazuo Yamanaka
