# Supplementary material for: Transcatheter aortic valve implantation versus conservative management for severe aortic stenosis in real clinical practice
Source: PLoS One. 2019 Sep 26;14(9):e0222979. doi: 10.1371/journal.pone.0222979 (PMC6762145; doi:10.1371/journal.pone.0222979)
Supplement: S3 Text — (DOCX) [file pone.0222979.s003.docx]

**S3 Text. List of relevant institutional review boards**

This study protocol was approved by all the institutional review board as follows:

**The institutional review board of Kyoto University Graduate School of Medicine.**

**The institutional review board of Kokura Memorial Hospital.**

**The institutional review board of Shimada Municipal Hospital.**

**The institutional review board of** **Shizuoka City Shizuoka Hospital.**

**The institutional review board of Kobe City Medical Center General Hospital.**

**The institutional review board of Kurashiki Central Hospital.**

**The institutional review board of Tenri Hospital.**

**The institutional review board of Nara Hospital, Kinki University Faculty of Medicine.**

**The institutional review board of Mitsubishi Kyoto Hospital.**

**The institutional review board of Kinki University Hospital.**

**The institutional review board of Kishiwada City Hospital.**

**The institutional review board of Osaka Red Cross Hospital.**

**The institutional review board of Koto Memorial Hospital.**

**The institutional review board of Shizuoka General Hospital.**

**The institutional review board of Nishikobe Medical Center.**

**The institutional review board of Japanese Red Cross Wakayama Medical Center.**

**The institutional review board of National Hospital Organization Kyoto Medical Center.**

**The institutional review board of The Tazuke Kofukai Medical Research Institute, Kitano Hospital.**

**The institutional review board of Hikone Municipal Hospital.**

**The institutional review board of Kansai Electric Power Hospital.**

**The institutional review board of Hyogo Prefectural Amagasaki General Medical Center.**

**The institutional review board of Rakuwakai Otowa Hospital.**

**The institutional review board of Saiseikai Noe Hospital.**

**The institutional review board of Shiga Medical Center for Adults.**

**The institutional review board of Hamamatsu Rosai Hospital.**

**The institutional review board of Japanese Red Cross Otsu Hospital.**

**The institutional review board of Hirakata Kohsai Hospital.**
