# Supplementary material for: Transcatheter aortic valve implantation versus conservative management for severe aortic stenosis in real clinical practice
Source: PLoS One. 2019 Sep 26;14(9):e0222979. doi: 10.1371/journal.pone.0222979 (PMC6762145; doi:10.1371/journal.pone.0222979)
Supplement: S4 Text — (DOCX) [file pone.0222979.s004.docx]

**S4 Text. Definitions of the Endpoints**

Death was regarded as cardiovascular in origin unless obvious non-cardiovascular causes could be identified. Sudden death was defined as unexplained death in previously stable patients. Any death during the hospitalization for aortic valve replacement or transcatheter aortic valve implantation was regarded as aortic valve procedure-related death. Aortic valve-related death included aortic valve procedure-related death, sudden death, and death due to heart failure possibly related to aortic stenosis. Heart failure hospitalization was defined as hospitalization due to worsening heart failure requiring intravenous drug therapy. Myocardial infarction (MI) during the follow-up period was defined in accordance with the universal definition of MI.^1^ Stroke was defined as ischemic or hemorrhagic stroke either requiring or prolonging hospitalization with symptoms lasting >24 hours. Life-threatening/disabling or major bleeding was defined as Bleeding Academic Research Consortium (BARC) classifications type 5, 3c, 3b, or 3a.^2^

Device success was defined that absence of aortic valve procedure death and correct positioning of a single prosthetic heart valve into the proper anatomical location and intended performance of the prosthetic heart valve (no prosthesis- patient mismatch and mean aortic valve gradient <20 mmHg or peak velocity >3 m/s, and no moderate or severe prosthetic valve regurgitation). ^2^. Major vascular complication was defined in the Valve Academic Research Consortium-2 (VARC-2) criteria. ^2^

**References**

1. Thygesen K, Alpert JS, White HD, Jaffe AS, Apple FS, Galvani M, et al. Universal definition of myocardial infarction. *Circulation*. 2007;116:2634-2653.

2. Kappetein AP, Head SJ, Genereux P, Piazza N, van Mieghem NM, Blackstone EH, et al. Updated standardized endpoint definitions for transcatheter aortic valve implantation: the Valve Academic Research Consortium-2 consensus document (VARC-2). *Eur J Cardiothorac Surg*. 2012;42:S45-60.
