# Supplementary material for: Transcatheter aortic valve implantation versus conservative management for severe aortic stenosis in real clinical practice
Source: PLoS One. 2019 Sep 26;14(9):e0222979. doi: 10.1371/journal.pone.0222979 (PMC6762145; doi:10.1371/journal.pone.0222979)
Supplement: S1 Fig — Distribution of propensity score in (A) the entire cohort and (B) PS matched cohort. (DOCX) [file pone.0222979.s005.docx]

**Supporting Figure titles and legends**

**S1 Figure. Distribution of propensity score in (A) the entire cohort and (B) PS matched cohort**

**S1 Figure**
