# Supplementary material for: Transcatheter aortic valve implantation versus conservative management for severe aortic stenosis in real clinical practice
Source: PLoS One. 2019 Sep 26;14(9):e0222979. doi: 10.1371/journal.pone.0222979 (PMC6762145; doi:10.1371/journal.pone.0222979)
Supplement: S5 Fig — Kaplan-Meier curves for (A) all-cause death and (B) heart failure hospitalization in the entire cohort. (DOCX) [file pone.0222979.s009.docx]

**Supporting Figure titles and legends**

**S5 Figure. Kaplan-Meier curves for (A) all-cause death and (B) heart failure hospitalization in the entire cohort**

**S5 Figure**
