# Supplementary material for: Transcatheter aortic valve implantation versus conservative management for severe aortic stenosis in real clinical practice
Source: PLoS One. 2019 Sep 26;14(9):e0222979. doi: 10.1371/journal.pone.0222979 (PMC6762145; doi:10.1371/journal.pone.0222979)
Supplement: S9 Fig — Subgroup analysis for the primary outcome measure: (A) All-cause moratality and (B) Heart failure hospitalization. (DOCX) [file pone.0222979.s013.docx]

**Supporting Figure titles and legends**

**S9 Figure. Subgroup analysis for the primary outcome measure: (A) All-cause mortality and (B) Heart failure hospitalization.**

**S9 Figure**
