# Supplementary material for: Transcatheter aortic valve implantation versus conservative management for severe aortic stenosis in real clinical practice
Source: PLoS One. 2019 Sep 26;14(9):e0222979. doi: 10.1371/journal.pone.0222979 (PMC6762145; doi:10.1371/journal.pone.0222979)
Supplement: S1 Table — (DOCX) [file pone.0222979.s014.docx]

**S1 Table. Coefficients of the independent variables in the logistic regression function**

| **Variable** | **coefficient** |
| --- | --- |
| Intercept | -2.2270 |
| Age ≥80 years | 1.7852 |
| Gender male | 0.5034 |
| Body mass index <22 kg/m^2^ | -0.7975 |
| Prior heart surgery | 1.2064 |
| Prior symptomatic stroke | -0.2312 |
| Creatinine level >2 mg/dl | -1.1136 |
| Anemia | 0.8575 |
| Malignancy | -0.4240 |
| Immunosuppressive therapy | 0.5592 |
| Chronic lung disease (moderate or severe) | 0.9719 |
| Vmax ≥5m/s | 0.7537 |
| LVEF <40% | -0.7203 |
| Any combined valvular disease (moderate or severe) | -1.5470 |

LVEF, left ventricular ejection fraction
