# Supplementary material for: Transcatheter aortic valve implantation versus conservative management for severe aortic stenosis in real clinical practice
Source: PLoS One. 2019 Sep 26;14(9):e0222979. doi: 10.1371/journal.pone.0222979 (PMC6762145; doi:10.1371/journal.pone.0222979)
Supplement: S2 Table — (DOCX) [file pone.0222979.s015.docx]

**S2 Table. Procedural characteristics and outcomes of the patients who underwent TAVI**

|  | **Entire cohort** | **PS matched cohort** |
| --- | --- | --- |
|  | **(N=449)** | **(N=278)** |
| **Procedural characteristics** |  |  |
| Approach site |  |  |
| Trans femoral | 282 (62.8%) | 183 (65.8%) |
| Trans apical | 146 (32.5%) | 84 (30.2%) |
| Trans iliac | 19 (4.2%) | 9 (3.2%) |
| Direct aorta | 2 (0.4%) | 2 (0.7%) |
| Procedure time (min) | 105.8± 51 | 105.4± 49 |
| 20-mm valve | 2 (0.4%) | 1 (0.4%) |
| 23-mm valve | 262 (58.4%) | 171 (61.5%) |
| 26-mm valve | 159 (35.4%) | 91 (32.7%) |
| 29-mm valve | 21 (4.7%) | 13 (4.7%) |
| General anesthesia | 444 (98.9%) | 276 (99.3%) |
| ECMO support | 14 (3.1%) | 9 (3.2%) |
|  |  |  |
| **Procedural outcomes** |  |  |
| Successful valve implantation | 437 (97.3%) | 273 (98.2%) |
| Device success | 413 (92.0%) | 257 (92.4%) |
| Annulus rupture | 3 (0.7%) | 2 (0.7%) |
| Conversion to open surgery | 4 (0.9%) | 2 (0.7%) |
| Coronary intervention | 3 (0.7%) | 1 (0.4%) |
| Major vascular complication | 20 (4.5%) | 13 (4.7%) |
| Pacemaker implantation | 20 (4.5%) | 13 (4.7%) |
| Hospital stay after TAVI (days) | 12 (9-18) | 12.5 (9-19) |

Categorical variables were presented as number (percentage), and continuous variables were presented as median (interquartile range).

Device success was defined that absence of aortic valve procedure death and correct positioning of a single prosthetic heart valve into the proper anatomical location and intended performance of the prosthetic heart valve (no prosthesis- patient mismatch and mean aortic valve gradient <20 mmHg or peak velocity >3 m/s, and no moderate or severe prosthetic valve regurgitation).

Successful valve implantation was defined absent of valve delivery failure, second valve implantation, annulus rupture and conversion to open heart surgery.

TAVI, transcatheter aortic valve implantation; PS, propensity score; ECMO, extra-corporeal membrane oxygenation.
