# Supplementary material for: Transcatheter aortic valve implantation versus conservative management for severe aortic stenosis in real clinical practice
Source: PLoS One. 2019 Sep 26;14(9):e0222979. doi: 10.1371/journal.pone.0222979 (PMC6762145; doi:10.1371/journal.pone.0222979)
Supplement: S3 Table — (DOCX) [file pone.0222979.s016.docx]

**S3 Table. Clinical outcomes at 30-day in the PS-matched cohort and in the entire cohort**

|  | **PS matched cohort** | |  | **Entire cohort** | |  |
| --- | --- | --- | --- | --- | --- | --- |
|  | TAVI group (N=278) | Conservative group (N=278) | Log-rank P | TAVI group (N=449) | Initial conservative group (N=984) | Log-rank P |
|  | N of Patients with Event | N of Patients with Event |  | N of Patients With Event | N of Patients With Event |  |
|  | (Cumulative 30-day incidence) | (Cumulative 30-day incidence) |  | (Cumulative 30-day incidence) | (Cumulative 30-day incidence) |  |
| All-cause death | 3 (1.1%) | 11 (4.1%) | 0.03 | 5 (1.1%) | 42 (4.3%) | 0.002 |
| Cardiovascular death | 3 (1.1%) | 9 (3.4%) | 0.07 | 5 (1.1%) | 35 (3.6%) | 0.008 |
| Aortic valve-related death | 3 (1.1%) | 6 (2.3%) | 0.28 | 5 (1.1%) | 23 (2.4%) | 0.11 |
| Aortic valve procedure death | 3 (1.1%) | 0 (0.0%) | 0.09 | 5 (1.1%) | 0 (0.0%) | 0.001 |
| Sudden death | 0 (0.0%) | 0 (0.0%) | - | 0 (0.0%) | 7 (0.7%) | 0.07 |
| Non-cardiovascular death | 0 (0.0%) | 2 (0.8%) | 0.15 | 0 (0.0%) | 7 (0.7%) | 0.07 |
| Heart failure hospitalization | 0 (0.0%) | 5 (1.9%) | 0.02 | 1 (0.2%) | 21 (2.1%) | 0.005 |
| Composite of aortic valve-related death or heart failure hospitalization | 3 (1.1%) | 10 (3.8%) | 0.04 | 6 (1.3%) | 42 (4.4%) | 0.003 |
| Myocardial infarction | 1 (0.4%) | 1 (0.4%) | 0.99 | 1 (0.2%) | 1 (0.1%) | 0.58 |
| Stroke | 5 (1.8%) | 1 (0.4%) | 0.11 | 10 (2.2%) | 7 (0.7%) | 0.02 |
| Major bleeding | 12 (4.3%) | 2 (0.8%) | 0.007 | 20 (4.5%) | 10 (1.1%) | <0.0001 |
| Infective endocarditis | 0 (0.0%) | 0 (0.0%) | - | 0 (0.0%) | 0 (0.0%) | - |

PS, propensity score; TAVI, transcatheter aortic valve implantation; N/A, not applicable.
