# Supplementary material for: Transcatheter aortic valve implantation versus conservative management for severe aortic stenosis in real clinical practice
Source: PLoS One. 2019 Sep 26;14(9):e0222979. doi: 10.1371/journal.pone.0222979 (PMC6762145; doi:10.1371/journal.pone.0222979)
Supplement: S4 Table — (DOCX) [file pone.0222979.s017.docx]

**S4 Table. Clinical outcomes in the entire cohort**

|  | **TAVI group** | **Conservative group** | **Hazard Ratio (95% Confidence Interval)** | | | |
| --- | --- | --- | --- | --- | --- | --- |
|  | **(N=449)** | **(N=984)** |  |  |  |  |
|  | N of Patients with Event | N of Patients with Event | Crude | P-value | Adjusted | P-value |
|  | (Cumulative 2-year incidence) | (Cumulative 2-year incidence) |  |  |  |  |
| All-cause death | 75 (17.3%) | 316 (33.9%) | 0.45 (0.35-0.60) | <0.0001 | 0.44 (0.33-0.59) | <0.0001 |
| Cardiovascular death | 34 (8.2%) | 238 (26.6%) | 0.27 (0.19-0.39) | <0.0001 | 0.27 (0.18-0.41) | <0.0001 |
| Aortic valve-related death | 13 (3.1%) | 172 (20.1%) | 0.15 (0.08-0.25) | <0.0001 | 0.13 (0.07-0.22) | <0.0001 |
| Aortic valve procedure death | 10 (2.3%) | 12 (1.6%) | 1.62 (0.68-3.75) | 0.27 | N/A | - |
| Sudden death | 9 (2.3%) | 46 (5.7%) | 0.38 (0.17-0.73) | 0.003 | 0.32 (0.14-0.69) | 0.003 |
| Non-cardiovascular death | 41 (9.8%) | 78 (10.0%) | 0.99 (0.67-1.44) | 0.97 | 0.89 (0.55-1.43) | 0.2 |
| Heart failure hospitalization | 40 (9.8%) | 277 (33.9%) | 0.25 (0.18-0.35) | <0.0001 | 0.24 (0.16-0.34) | <0.0001 |
| Composite of aortic valve-related death or heart failure hospitalization | 50 (11.9%) | 341 (39.1%) | 0.26 (0.19-0.35) | <0.0001 | 0.25 (0.18-0.34) | <0.0001 |
| Myocardial infarction | 1 (0.2%) | 11 (1.5%) | 1.00 (0.05-5.14) | 1 | 0.36 (0.02-2.45) | 0.33 |
| Stroke | 22 (5.4%) | 38 (5.0%) | 1.14 (0.67-1.91) | 0.62 | 1.09 (0.56-2.12) | 0.79 |
| Major bleeding | 42 (9.8%) | 47 (6.0%) | 1.84 (1.21-2.79) | 0.005 | 1.68 (0.98-2.88) | 0.06 |
| Infective endocarditis | 6 (1.5%) | 3 (0.4%) | 3.81 (1.01-18.1) | 0 | N/A | - |

TAVI, transcatheter aortic valve implantation; N/A, not applicable.
