# Supplementary material for: Transcatheter aortic valve implantation versus conservative management for severe aortic stenosis in real clinical practice
Source: PLoS One. 2019 Sep 26;14(9):e0222979. doi: 10.1371/journal.pone.0222979 (PMC6762145; doi:10.1371/journal.pone.0222979)
Supplement: S5 Table — (DOCX) [file pone.0222979.s018.docx]

**S5 Table. Clinical outcomes in the PS-matched cohort after excluding those patients who died within 30 days after the index echocardiography in the conservative group**

|  | **TAVI group** | **Conservative group** | **Hazard Ratio (95% Confidence Interval)** | | | |
| --- | --- | --- | --- | --- | --- | --- |
|  | **(N=267)** | **(N=267)** |  |  |  |  |
|  | N of Patients with Event | N of Patients with Event | Crude | P-value | Adjusted | P-value |
|  | (Cumulative 2-year incidence) | (Cumulative 2-year incidence) |  |  |  |  |
| All-cause death | 42 (16.3%) | 84 (33.9%) | 0.44 (0.30-0.63) | <0.0001 | 0.49 (0.33-0.74) | 0.0006 |
| Cardiovascular death | 19 (7.7%) | 60 (25.5%) | 0.28 (0.16-0.46) | <0.0001 | 0.29 (0.16-0.49) | <0.0001 |
| Aortic valve-related death | 5 (2.0%) | 48 (21.2%) | 0.09 (0.03-0.21) | <0.0001 | 0.09 (0.03-0.21) | <0.0001 |
| Aortic valve procedure death | 4 (1.5%) | 2 (1.0%) | 1.82 (0.36-13.1) | 0.48 | N/A | - |
| Sudden death | 6 (2.5%) | 15 (6.9%) | 0.35 (0.13-0.87) | 0.02 | N/A | - |
| Non-cardiovascular death | 23 (9.3%) | 24 (11.3%) | 0.84 (0.47-1.49) | 0.54 | 1.11 (0.58-2.17) | 0.75 |
| Heart failure hospitalization | 25 (10.3%) | 84 (37.0%) | 0.24 (0.15-0.37) | <0.0001 | 0.24 (0.15-0.38) | <0.0001 |
| Composite of aortic valve-related death or heart failure hospitalization | 29 (11.8%) | 97 (41.1%) | 0.24 (0.16-0.36) | <0.0001 | 0.23 (0.15-0.36) | <0.0001 |
| Myocardial infarction | 0 (0%) | 2 (1.1%) | N/A | - | N/A | - |
| Stroke | 11 (4.5%) | 11 (5.3%) | 0.90 (0.38-2.10) | 0.8 | N/A | - |
| Major bleeding | 24 (9.4%) | 12 (5.3%) | 1.92 (0.98-3.98) | 0.06 | N/A | - |
| Infective endocarditis | 6 (2.5%) | 1 (0.5%) | 5.40 (0.92-101.9) | 0.06 | N/A | - |

PS, propensity score; TAVI, transcatheter aortic valve implantation; N/A, not applicable.
